# Geotricosis: fungemia en paciente con leucemia linfoblástica aguda

**DOI:** 10.7705/biomedica.6779

**Published:** 2023-08-31

**Authors:** José Camilo Álvarez-Rodríguez, María Paula Blanco-Bustos, Sonia Isabel Cuervo-Maldonado, Julio César Gómez-Rincón, Ángela Reyes

**Affiliations:** 1 Grupo en Enfermedades Infecciosas en Cáncer y Alteraciones Hematológicas, Universidad Nacional de Colombia, Bogotá, D.C., Colombia Universidad Nacional de Colombia Universidad Nacional de Colombia Bogotá D.C Colombia; 2 Grupo de Infectología, Instituto Nacional de Cancerología E.S.E, Bogotá, D.C., Colombia Instituto Nacional de Cancerología E.S.E Bogotá D.C Colombia; 3 Facultad de Medicina, Universidad Nacional de Colombia, Bogotá, D.C., Colombia Universidad Nacional de Colombia Universidad Nacional de Colombia Bogotá D.C Colombia; 4 Grupo de Microbiología, Laboratorio Clínico, Instituto Nacional de Cancerología E.S.E, Bogotá, Colombia Instituto Nacional de Cancerología E.S.E Bogotá Colombia

**Keywords:** geotricosis, fungemia, leucemia-linfoma linfoblástico de células precursoras, anfotericina B, voriconazol, espectrometría de masa por láser de matriz asistida de ionización desorción, Geotrichosis, fungemia, precursor cell lymphoblastic leukemialymphoma, amphotericin B, voriconazole, spectrometry, mass, matrix-assisted laser desorption-ionization

## Abstract

La fungemia por *Geotrichum* spp. es poco frecuente y altamente letal. En el Instituto Nacional de Cancerología de Bogotá solo se han reportado dos casos: uno entre el 2001 y el 2007, y el otro entre el 2012 y el 2018. Este tipo de infección es más común en pacientes con algún grado de compromiso del sistema inmunitario, por lo que puede presentarse en pacientes con neoplasias hematológicas malignas.

Se presenta el caso de un hombre de 27 años con recaída de leucemia linfoblástica aguda, que ingresó con poliartralgias de cinco días de duración. También cursaba con neutropenia febril, celulitis sin abscesos y bacteriemia por *Staphylococcus aureus* resistente a la meticilina para lo cual recibió terapia con oxacilina y cefepime. Sin embargo, persistía la neutropenia febril por lo que se sospechó una infección fúngica invasora. Se tomó un nuevo set de hemocultivos y se inició tratamiento antifúngico.

En los hemocultivos se identificaron artroconidias y mediante espectrometría de masas por láser de matriz asistida de ionización-desorción se confirmó la presencia de *Geotrichum* spp. Se ajustó el tratamiento antifúngico con deoxicolato de anfotericina B por 14 días *y* voriconazol por cuatro semanas. Luego de una estancia prolongada se le dio de alta. Aunque la incidencia de la fungemia por *Geotrichum* spp. es baja*,* en pacientes con neoplasias hematológicas malignas debe considerarse en el contexto de una neutropenia febril que es persistente a pesar del tratamiento antimicrobiano de amplio espectro. La identificación de los agentes causantes de fungemias con herramientas de proteómica, como la espectrometría de masas mencionada, permite ajustar el tratamiento dirigido y reducir las complicaciones, la estancia hospitalaria y la mortalidad.

## Abstract (en)

Fungemia caused by *Geotrichum* spp. is rare and highly lethal. The *Instituto Nacional de Cancerología* in Bogotá reported just two cases: one in the period 2001-2007 and the other in 2012-2018. This type of infection is more common in any kind of immunocompromised patients, so it can occur in those with hematological malignancies.

Here we present the case of a 27-year-old man, diagnosed with acute lymphoblastic leukemia in relapse and admitted with polyarthralgia for five days, febrile neutropenia, non- abscessed cellulitis, and bacteremia due to methicillin-sensitive *Staphylococcus aureus.* The patient received therapy with oxacillin and cefepime, but the febrile neutropenia persisted. A new set of blood cultures was taken, and antifungal treatment was started because of the suspicion of invasive fungal infection. Arthroconidia were identified in blood cultures and *Geotrichum* spp. was confirmed using matrix-assisted laser desorption-ionization mass spectrometry. The antifungal treatment was adjusted with amphotericin B deoxycholate for 14 days and voriconazole for four weeks, and after a prolonged stay, the patient was discharged. Although the incidence of fungemia caused by *Geotrichum* spp. is low, it must be considered in patients with hematological malignancies and persistent febrile neutropenia despite the broadspectrum antimicrobial treatment. The confirmation of fungemia causing agents, with proteomic tools such as the mentioned mass spectrometry, allows treatment adjustment and decreases complications, hospital stay, and mortality.

La fungemia por *Geotrichum* spp. es poco frecuente y altamente letal. En el Instituto Nacional de Cancerología de Bogotá solo se han presentado dos casos: uno entre los años 2001 a 2007 (Cortés JA, Cuervo Si, Rivas P. Fungemia por hongos diferentes a *Candida* spp. en pacientes con cáncer en el Instituto Nacional de Cancerología, 1999-2006. En: V Encuentro Nacional de Investigación en Enfermedades Infecciosas, Armenia, 2006. Bogotá: Asociación Colombiana de Infectología; 2006. p.104) y el otro, en el periodo comprendido entre el 2012 y el 2018 en el estudio realizado en varias instituciones prestadoras de servicios oncológicos de Bogotá ^1^. En la serie de casos reportados por Chitasombat *et al.*, *Geotrichum* spp. representa el cuarto agente patógeno en frecuencia (5 % de los casos), con un cociente de riesgo de mortalidad de 111,3 (9,9-1255,2). Los dos aislamientos fueron sensibles a los azoles, resistentes a las equinocandinas y uno resistente a la anfotericina B ^2^.

## Caso clínico

Se presenta el caso de un hombre de 27 años, procedente de Bogotá, vendedor de autopartes, con diagnóstico de leucemia linfoblástica aguda de precursores de células B, de tipo común CD20+, de alto riesgo. Recibió dos ciclos de HyperCVAD y consultó al Instituto Nacional de Cancerología por recaída y poliartralgias de cinco días de evolución, por lo que se le inició el protocolo establecido en el 2003 por el Grupo de Investigación de Leucemia Linfoblástica Aguda en Adultos (GRAALL-2003).

Posteriormente, el paciente cursó con neutropenia febril, celulitis en la mano izquierda -sin abscesos- y bacteriemia por *Staphylococcus aureus* sensible a la meticilina. Se le administró cefapime y oxacilina y se resolvió la bacteriemia.

En el día 21 de hospitalización, el paciente presentó lesiones en el dorso de la mano izquierda, de tipo placa eritematoedematosa, con descamación leve y fina en la superficie y costra hemática central, así como lesiones en la región escapular e infraescapular derecha con presencia de pápulas de 3 mm, no foliculares, agrupadas, algunas eritematosas y otras eritematoparduzcas, sin cambio epidérmico. Las lesiones fueron valoradas por el Servicio de Dermatología y dadas las características de aquellas en el dorso de la mano se tomó biopsia de piel para estudio histopatológico. Los resultados no fueron concluyentes para el diagnóstico de micosis y no se hizo cultivo para hongos de la biopsia.

Debido a la persistencia de la neutropenia febril y a la aparición de las lesiones en piel, se sospechó una infección fúngica invasora, por lo que se inició tratamiento con caspofungina. Las tomografías computarizadas (TC) de tórax y de senos paranasales, así como la detección de galactomanano sérico fueron negativas para aspergilosis invasora.

No obstante, a pesar del tratamiento antimicrobiano y antifúngico, el paciente no presentaba mejoría significativa, ya que persistía con fiebre hasta de 40 °C, sin otros síntomas localizadores. El paciente continuó en regular estado general, somnoliento, con palidez mucocutánea leve y con nódulos de 1 cm, blandos, depresibles, indoloros y en los sitios previos de venopunción, sin otros hallazgos de importancia.

Se decidió tomar nuevas muestras para hemocultivo y se solicitó otra biopsia de las lesiones cutáneas. El hemograma evidenció una neutropenia profunda (20 glóbulos blancos por µl) y linfopenia persistente; la proteína C reactiva fue de 6,15 mg/dl, la albúmina estaba en 1,94 g/dl y la creatinina y las transaminasas fueron normales. En la TC de control de senos paranasales se evidenciaron cambios inflamatorios crónicos incipientes en el seno maxilar derecho; la TC de tórax fue normal, pero en la de abdomen se documentó hepatoesplenomegalia.

**Figura 1:**
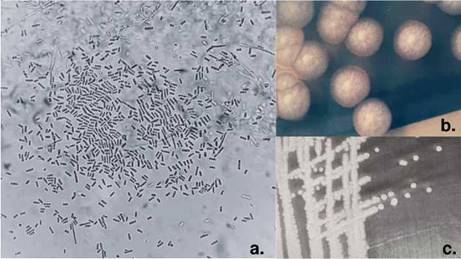
a. Hemocultivo de *Geotrichum* spp., se observan artroconidias hialinas rectangulares, Gram, 100X. b. En el cultivo se observa una colonia de aspecto algodonoso, de color blanco o crema. c. Colonias de *Geotrichum* spp. en agar Sabouraud.

En los tres hemocultivos y en el urocultivo del día 28 de hospitalización se visualizaron estructuras levaduriformes, no identificadas en el panel automatizado Yeast ID (BD Phoenix™ 100). Por microscopía se observaron artroconidias y se documentaron colonias compatibles con *Geotrichum* spp. (figura 1), diagnóstico confirmado por espectrometría de masas por láser de matriz asistida de ionización desorción (MALDI-TOF).

Se formuló tratamiento con deoxicolato de anfotericina B en una dosis diaria de 1 mg/kg por 14 días y 400 mg/día de voriconazol por cuatro semanas. Los síntomas clínicos se resolvieron y el paciente se recuperó de la neutropenia en el día 24 del tratamiento antifúngico combinado. Por mejoría clínica, el paciente egresó en el día 101 de hospitalización.

## Consideraciones éticas

La descripción del caso clínico corresponde a uno de los casos registrados en el proyecto de investigación “Fungemia en pacientes con neoplasias hematológicas y neutropenia febril postquimioterapia 2012-2018, en instituciones de Bogotá”, aprobado por el Comité de Ética de Investigaciones del Instituto Nacional de Cancerología, identificado con el código C1901300473.

## Discusión

El género *Geotrichum* está conformado por 108 especies, pertenece al filo *A* scomycota, orden Saccharomycetales, familia Diposdascaceae ^3^ y forma artroconidias. El estadio sexual de *Geotrichum candidum* es *Galactomyces candidus*, que inicialmente se pensó que era una especie diferente, pero por secuenciación se confirmó que no era así. *Geotrichum candidum* se encuentra en la tierra, en los detritos vegetales y en la arena de playa; también es utilizado en la fabricación de productos lácteos como quesos y yogurt, entre otros ^4^. En el ser humano puede colonizar el sistema digestivo, la cavidad oral, el sistema respiratorio, la piel y el cabello ^5^. Las especies que se consideran potencialmente patógenas para el ser humano son *G. candidum*, *G. capitatum y G. clavatum*^4^.

La identificación de *Geotrichum* spp. se hace por las características macroscópicas de las colonias -que son blanco-amarillentas- y por los aspectos bioquímicos: *G. capitatum* puede crecer en presencia de cicloheximida mientras que *G. candidum* no puede hacerlo ^5^. *Geotrichum* spp*.* se diferencia de *Trichosporon* spp*.* por la ausencia en la detección de actividad de la enzima ureasa y porque necesita una temperatura óptima de crecimiento de 30 ^o^C ^6^. Por microscopía se observa que las hifas de las especies de *Geotrichum* se dividen en artrosporas alargadas, por lo que se confunde fácilmente con *Trichosporon* spp. ^7^, dificultando su identificación por medio de los sistemas automatizados. Sin embargo, los análisis proteómicos mediante MALDITO-F permiten diferenciar *Geotrichum* spp. de otros ascomicetos y basidiomicetos ^8^^,^^9^.

En los pacientes inmunosuprimidos, la infección fúngica invasora por lo general ocurre por hongos de los géneros *Candida y Aspergillus*, aunque también por otros emergentes como *Geotrichum*^10^, que es el caso del presente reporte clínico. Estas infecciones son frecuentes en pacientes con neoplasias hematológicas malignas, neutropenia y trasplante alogénico de progenitores hematopoyéticos ^7^. A pesar de su baja frecuencia, esta infección fúngica causa alta mortalidad principalmente en los casos de leucemia mieloide aguda ^11^. En la revisión de Gao *et al.*, se describen, en un periodo de 46 años (1965 a 2011), 202 casos de infección fúngica invasora con mortalidad del 50 % ^7^. Esto es semejante a lo que se presenta en el Instituto Nacional de Cancerología, en donde en un periodo de siete años sólo se encontró un caso de infección fúngica invasora por *Geotrichum* spp.

La infección fúngica invasora por especies de *Geotrichum* también se ha descrito en casos de pacientes con diversos factores de riesgo como el tratamiento con corticoides o antibióticos de amplio espectro, el uso de dispositivos vasculares, la alimentación parenteral y la alteración localizada de la inmunidad por alteración de la piel y las mucosas (úlceras) ^10^^-^^12^.

El registro de infecciones fúngicas invasoras, causadas por levaduras y hongos filamentosos emergentes, ha aumentado porque los laboratorios clínicos disponen de nuevas herramientas diagnósticas para su identificación como los métodos automatizados y proteómicos como MALDITO-F ^13^. En las Américas, las tasas de morbilidad y mortalidad por infección fúngica invasora están entre el 65 y el 90 % en el grupo de pacientes con neoplasias hematológicas malignas ^14^.

Dado que la presentación clínica de la infección por levaduras y otros hongos emergentes se puede confundir con la candidiasis invasora ^14^, usualmente se prescribe un tratamiento empírico para *Candida* spp*.* Sin embargo, este tratamiento es inefectivo para los organismos emergentes ya que tienen un comportamiento agresivo y su diagnóstico tardío junto con un tratamiento inadecuado puede ser fulminante ^14^. En la revisión del registro de FungiScope desde 1976 hasta 2016, en Colombia no se reportaron casos de infección fúngica invasora por *Geotrichum* spp. o *Saprochaete* spp. En Latinoamérica, existe registro hasta el año 2016 de uno a diez casos en Argentina, y uno en Brasil ^15^. En el Instituto Nacional de Cancerología, como ejemplo de los casos descritos en Colombia sobre infección fúngica invasora por *Geotrichum,* solo se ha informado un caso entre los años 2001 y 2007 ^16^.

Las rutas de infección son por vía respiratoria, digestiva, gastrointestinal o tegumentaria, especialmente en pacientes con leucemias agudas linfoides o mieloides, con una tasa de infección de 0,5 % ^11^. La mortalidad que se informa en estudios multicéntricos es mayor del 50 %. En pacientes con neoplasia hematológica maligna y neutropenia las manifestaciones clínicas son similares a las de otras infecciones fúngicas y es difícil diferenciarlas por la clínica del agente causal ^14^. La mayoría presenta fiebre, a pesar del tratamiento con antimicrobianos de amplio espectro, compromiso de diferentes órganos y pueden aparecer lesiones cutáneas máculo-papulares generalizadas como las que tuvo el paciente de este caso. Según otros reportes clínicos, también se manifiestan como lesiones pápulo-vesiculares o purpúricas que pueden afectar mucosas como la de la faringe ^4^; el compromiso de la vía aérea puede ser bronquial o pulmonar, caracterizado por tos, dolor pleurítico, derrame pleural y neumotórax espontáneo; en el sistema digestivo puede afectarse la boca, el intestino, el hígado y el bazo, manifestaciones similares a las de la candidiasis crónica diseminada. También se ha descrito compromiso ótico.

Los factores de riesgo descritos en la literatura para el desarrollo de una infección fúngica invasora incluyen neutropenia, inmunodeficiencia, dispositivos vasculares permanentes, tratamiento con agentes inmunosupresores, quimioterapia y antibióticos de amplio espectro ^1^^,^^17^. El control de estos factores de riesgo es una estrategia adecuada para reducir la tasa de mortalidad que, no obstante, sigue siendo alta. En una serie de 26 casos, se reportó el 52 % de mortalidad. Los pacientes presentaron una mejoría en la supervivencia cuando se les retiraron los dispositivos vasculares centrales. Esto evidenció que en un paciente con buen estado funcional y con terapia antifúngica empírica o profiláctica se puede reducir la probabilidad de mortalidad ^18^. Por otro lado, García-Ruiz y colegas reportaron una serie de cinco de casos de infección por *G. capitatum*: cuatro en pacientes con leucemia aguda y uno con linfoma de Burkitt. Los pacientes presentaban neutropenia profunda y prolongada, y estaban bajo un esquema de quimioterapia de inducción o rescate. Su mortalidad fue del 60 % ^19^ dada la persistencia de factores de riesgo como la neutropenia y la quimioterapia.

Para el diagnóstico etiológico de *Geotrichum* spp., la primera herramienta por mencionar es la histopatología, útil en la descripción del tipo de infiltrado y la identificación de la morfología y el tamaño de las hifas mediante la tinción de Grocott-Gomori a base de plata con metenamina ^17^. No obstante, existen características fenotípicas compartidas con otras especies de hongos que no permiten determinar puntualmente el agente etiológico. Por otro lado, el hemocultivo usualmente es positivo en el 70 % de los pacientes con enfermedad por *Geotrichum* spp*.,* mientras que para *Candida* spp*.* es menor del 50 %. También se puede identificar por medio de un cultivo de esputo y en el caso de diseminación cutánea se puede evaluar con los hallazgos histopatológicos y el cultivo de piel en medio específico para hongos. Cuando el compromiso es pulmonar, en la TC se pueden observar nódulos con aumento de la densidad de los tejidos blandos.

Las pruebas inmunológicas para identificar anticuerpos dirigidos contra moléculas de la pared del hongo, como el 1,3 beta-D-glucano y el galactomanano, son de uso frecuente en pacientes inmunosuprimidos como aquellos con neoplasias hematológicas malignas. El 1,3 beta-D-glucano es usado cuando se sospechan infecciones por *Aspergillus* spp*.* y *Candida* spp*.,* pero tiene poco impacto para identificar especies de *Geotrichum* spp. El galactomanano se recomienda como parte de la estrategia para la prescripción de tratamiento antifúngico anticipado, aunque un resultado positivo puede indicar la presencia de otros hongos. El seguimiento en el tiempo de los resultados de esta prueba es más aplicable para detectar infección por *Aspergillus* spp*.* y puede ser de utilidad en infecciones por *Geotrichum* spp. ^20^. El interés por identificar la especie se fundamenta en su utilidad para la selección del tratamiento antifúngico adecuado, por lo que la aplicación de la espectrometría de masas MALDI-TOF es de gran utilidad en estos casos ^17^.

Para el tratamiento se debe tener en cuenta la sensibilidad *in vitro* de *Geotrichum* spp. para anfotericina B, flucitosina, fluconazol, itraconazol y voriconazol ^21^. La concentración inhibitoria mínima de fluconazol va de 16 a 32 pg/ml, siendo categorizada por el *National Committee for Clinical Laboratory Standards* como sensible dependiendo de la dosis. Varias cepas fueron sensibles *in vitro* a la flucitosina, aunque su toxicidad hematológica hace que este antifúngico sea poco atractivo para el tratamiento en pacientes con neoplasias hematológicas malignas. Además, el voriconazol muestra una alta actividad *in vitro* comparable al itraconazol ^22^. Pocos estudios clínicos describen la sensibilidad de *Geotrichum* spp. a los antifúngicos; Miglietta *et al*. describen en un caso clínico de *G. capitatum,* una concentración inhibitoria mínima de 0,5 µg/ml para anfotericina B y voriconazol; de 0,12 µg/ml para voriconazol e itraconazol; de 2 µg/ml para anidulafungina; de 8 µg/ml para caspofungina y micofungina, y de 16 g/ml para flucitosina y fluconazol ^17^.

Los reportes de caso sugieren que la eficacia de la anfotericina B y el voriconazol es mayor que la reportada para el fluconazol, el itraconazol o la flucitosina ^4^^,^^7^, tal y como lo confirma el caso clínico en cuestión, donde el paciente logró control de la infección fúngica invasora con el tratamiento mencionado. La ventaja de esta combinación también se evidenció en un grupo de 13 pacientes, de los cuales 12 se curaron con el uso de la anfotericina B: cinco fueron tratados con anfotericina en monoterapia, mientras que los restantes recibieron una combinación de anfotericina B con otro antifúngico ^23^. En otra serie de casos, a uno de los 6 pacientes reportados se le suministró terapia combinada de anfotericina B y voriconazol, siendo el único sobreviviente, junto con otro paciente que recibió monoterapia con fluconazol ^4^^,^^24^.

La disponibilidad de nuevos antifúngicos, como el posaconazol y el isavuconazol, en la profilaxis antifúngica en pacientes con alto riesgo de presentar infección invasora puede ser una estrategia efectiva para disminuir su incidencia ^7^^,^^24^. El isavuconazol tiene una acción potente *in vitro* frente a especies atípicas de levaduras como *Trichosporon*, *Saccharomyces*, *Geotrichum*, *Pichia y Rhodotorula* con valores de concentración inhibitoria mínima similares al del voriconazol y el posaconazol ^24^. De igual forma, el posaconazol no está directamente indicado para *Geotrichum* spp., aunque es un antifúngico que ha demostrado tener una concentración mínima inhibitoria semejante a la del isavuconazol para esta levadura, por lo que puede ser una alternativa terapéutica cuando se requiera ^24^^,^^25^.

El tratamiento recomendado por la *European Society of Clinical Microbiology and Infectious Diseases* y, posteriormente, por la *European Confederation of Medical Mycology* para la fungemia por *Geotrichum* spp*.* es la anfotericina B en liposomas, que se prefiere sobre la no liposómica ya que esta última presenta mayor actividad nefrotóxica ^12^. También se recomienda el deoxicolato de anfotericina B con flucitosina o sin ella, así como la terapia con voriconazol ^26^^,^^27^, con la que se ha logrado una experiencia terapéutica positiva ^28^, evidenciada en este reporte de caso.

Por otro lado, no se recomienda el uso de equinocandinas (caspofungina o micafungina) ni de fluconazol, ya que los valores altos de sus concentraciones inhibitorias mínimas para *Geotrichum* spp*.* y *Trichosporon* spp. sugieren una pobre respuesta terapéutica; en caso de que el paciente esté en tratamiento con alguno de estos antifúngicos sin presentar mejoría, podría aumentar la sospecha de infección fúngica invasora por hongos naturalmente resistentes a equinocandinas, como por ejemplo, *Geotrichum* spp. ^24^.

En conclusión, la infección por *Geotrichum* spp*.* en pacientes con neoplasias hematológicas malignas, especialmente aquellos con leucemia aguda, genera una alta probabilidad de mortalidad. Por lo tanto, la búsqueda de microorganismos oportunistas emergentes como *Geotrichum* spp. en pacientes con malignidad hematológica, sin respuesta al tratamiento antimicrobiano, como a equinocandinas, permitiría sospechar de una infección por hongos y levaduras emergentes.

La identificación de los agentes etiológicos infecciosos requiere, además de la sospecha clínica, del uso de diferentes tipos de herramientas como hemocultivos, pruebas para la detección de lipopolisacáridos de hongos (galactomanano), análisis proteómicos, imágenes de tomografía y estudios histopatológicos. Una correcta identificación de la especie patógena permitirá prescribir un tratamiento antifúngico adecuado que, en conjunto con la recuperación de la neutropenia en los pacientes con leucemia aguda, puede tener un impacto positivo en la supervivencia de este grupo de pacientes.
